# Avian cardiomyocyte architecture and what it reveals about the evolution of the vertebrate heart

**DOI:** 10.1098/rstb.2021.0332

**Published:** 2022-11-21

**Authors:** Holly A. Shiels

**Affiliations:** Division of Cardiovascular Sciences, Faculty of Biology Medicine and Health, University of Manchester, Manchester, UK

**Keywords:** bird, sarcoplasmic reticulum, calcium release units, endothermy, ploidy, proliferation

## Abstract

Bird cardiomyocytes are long, thin and lack transverse (t)-tubules, which is akin to the cardiomyocyte morphology of ectothermic non-avian reptiles, who are typified by low maximum heart rates and low pressure development. However, birds can achieve greater contractile rates and developed pressures than mammals, whose wide cardiomyocytes contain a dense t-tubular network allowing for uniform excitation–contraction coupling and strong contractile force. To address this apparent paradox, this paper functionally links recent electrophysiological studies on bird cardiomyocytes with decades of ultrastructure measurements. It shows that it is the strong transsarcolemmal Ca^2+^ influx via the L-type Ca^2+^ current (*I*_CaL_) and the high gain of Ca^2+^-induced Ca^2+^ release from the sarcoplasmic reticulum (SR), coupled with an internal SR Ca^2+^ release relay system, that facilitates the strong fast contractions in the long thin bird cardiomyocytes, without the need for t-tubules. The maintenance of an elongated myocyte morphology following the post-hatch transition from ectothermy to endothermy in birds is discussed in relation to cardiac load, myocyte ploidy, and cardiac regeneration potential in adult cardiomyocytes. Overall, the paper shows how little we know about cellular Ca^2+^ dynamics in the bird heart and suggests how increased research efforts in this area would provide vital information in our quest to understand the role of myocyte architecture in the evolution of the vertebrate heart.

This article is part of the theme issue ‘The cardiomyocyte: new revelations on the interplay between architecture and function in growth, health, and disease’. Please see glossary at the end of the paper for definitions of specialized terms.

## 1. Introduction

**Figure 1.**
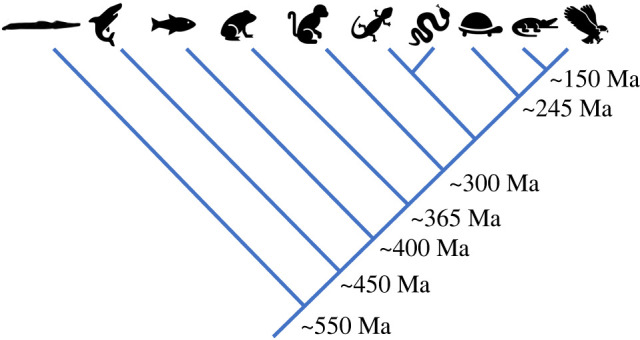
Schematic of the vertebrate phylogeny with taxa from left to right as follows: jawless fishes, cartilaginous fishes, teleost fishes, amphibians, mammals, lizards, snakes, turtles, crocodilians, birds. Numbers are estimated the time since last common ancestor; Ma is million years ago. Adapted from [2–5].

Birds and mammals evolved independently approximately 300 Ma from reptile-like ancestors [1] (figure 1) and both classes have acquired high resting metabolic rates and endothermy through convergent evolution. The evolutionary processes that have led to endothermy in birds and mammals are a matter of active debate [6–9] including the recent suggestion that whole body endothermy emerged across multiple and diverse taxa as by-product of energy balance regulation [10]. Regardless of the evolutionary driver(s) for endothermy in birds and mammals, a powerful heart is required to satisfy the high metabolic rates dictated by endothermy [11]. A powerful heart can deliver high volumes of oxygenated blood to the respiring tissues and provide the pressure necessary to drive filtration at the kidneys, linking the convergent evolution of the four-chambered heart and endothermy in birds and mammals. The anatomical separation of left and right ventricles in birds and mammals allows the elevation of systemic pressure significantly above pulmonary pressure thereby providing the necessary convection for highly aerobic tissues, whilst avoiding the rupture of thin respiratory surfaces [12–14]. The presence of a specialized conduction system [15] and a compact atrial and ventricular wall architecture is important for the fast atrioventricular conduction and rapid ventricular repolarization [16,17] of avian and mammalian hearts compared with those of ectothermic vertebrates (independent of temperature) [14,15,18]. Indeed, the convergent evolution of rapid/early repolarization in birds (zebrafinch) and mammals (mouse) is undoubtably important for achieving the fast heart rates typical of endotherms [17].

Despite these similarities between mammalian and avian hearts, there are also differences. When comparing animals of similar body size, birds have a nearly twofold larger heart mass than mammals [19–21]. This increases stroke volume allowing birds to pump more blood per unit time than mammals [22,23]. Birds also have elevated systolic and diastolic blood pressures compared with similarly sized mammals [20] meaning that stroke work (the product of stroke volume and blood pressure) is higher in birds than mammals [11,21]. Thus, within the two vertebrate groups demonstrating whole body endothermy, on average the bird heart is capable of equal or greater output than the mammalian heart, a feature which may be necessary to support their elevated body temperature (average bird 41°C, average placental mammal 37°C [22]) and the energetic costs of flight [7,19,24,25].

**Figure 2.**
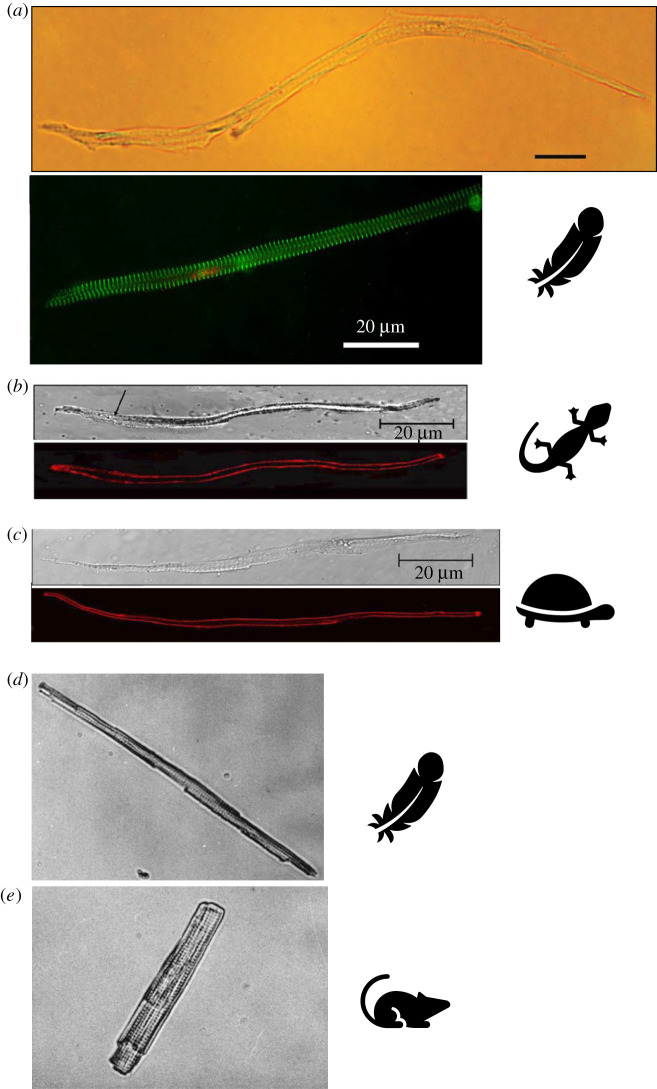
Images of freshly isolated ventricular myocytes from (*a*) Japanese quail *Coturnix japonica* as light microscope image (top) [32] and an immunofluorescent image with sarcomeres delineated with a green probe to α-actinin and nucleus in red (bottom) [16], (*b*) varanid lizard *Varanus exanthematicus* light microscope image, arrow is pointing to sarcomeric striations (top) and confocal image with the sarcolemmal membrane visible in red (bottom) [30], (*c*) yellow-bellied turtle *Trachemys scripta scripta* light microscope image (top) and confocal image with the sarcolemmal membrane visible in red (bottom) [29]. Photomicrograph image of a finch (*d*) and rat (*e*) cardiomyoctyte used with permission from [36]. In each image the vertical height of the image is 200 µm. Scale bar in all other images is 20 µm. (Online version in colour.)

Considering the robust cardiac performance of the bird heart, it is perhaps surprising that the gross morphology of the cardiomyocytes from which is it comprised, more closely resemble those of their non-avian reptilian ancestors than those of mammals (table 1 and figure 2). Cardiomyocytes of adult bird hearts are long (greater than 100 µm) and thin (less than 10 µm) with a small cross-sectional area (approx. 56 µm^2^) and a small cell volume (approx. 10 pl), leading to a large surface-area-to-volume ratio [31,32,36–38] (table 1). This morphology is similar to the cardiomyocytes of non-avian reptiles, amphibians and fish (table 1, and see [39]). This spindle/elongated myocyte morphology (figure 2) is considered ‘sufficient’ for powering the lower heart rates and lower blood pressures associated with ectothermic taxa [40]. The gross morphology of adult mammalian ventricular cardiomyocytes is unique across vertebrates. They are shorter (less than 100 µm) and wider (approx. 25 µm) and contain a network of transverse (t)-tubules which coordinate and synchronize excitation–contraction coupling across the entire volume of these wider cells [41] (figure 2). This ‘brick-like’ morphology develops postnatally with neonatal mammalian ventricular myocytes conforming to the elongated morphology of ectothermic taxa and then transitioning to the hypertrophied adult form soon after birth [42] (also see the contribution by Birkedal *et al.* [43] in this special issue). The hypertrophied adult ventricular myocyte morphology is thought to underpin the fast and strong contractions required to power the adult mammalian heart [44]. How then, do the long, thin, non-tubulated myocytes, characteristic of the slow and low-powered hearts of ectotherms, drive the enhanced cardiac performance of birds? This paper will first review the ultrastructure literature which predicts reconciliation of this apparent paradox by the remodelling of the subcellular organization of calcium (Ca^2+^) release units (CRUs) within the avian compared with ectotherm myocyte. The paper will then discuss the limited functional data on cellular Ca^2+^ dynamics from adult birds to form a working structure–function schema for bird excitation–contraction coupling. Finally, other traits associated with myocyte architecture will be discussed including volume regulation of cardiac output, and the apparent trade-off between myocyte proliferation-potential and polyploidy with cardiac growth. Rather than being definitive, the paper highlights how little we know about cellular Ca^2+^ dynamics in the bird heart and why research in this area is important to understand the role of myocyte architecture in the evolution of the vertebrate heart.

**Table 1.** Comparative morphometric data for vertebrate ventricular myocytes.

	lamprey^a^	zebrafish^b^	frog	rat^c^	turtle^d^	lizard^e^	snake^f^	alligator^g^	turkey^h^	quail^i^
cell length (μm)	323	100	300^j^	141.9	189.1	151.2	—	140	136	179.3
cell width (μm)	11.9	4.6	5^j^	32.0	7.2	5.9	—	5	8.7	8.3
cell depth (μm)	—	6.0	—	13.3	5.4	5.6	—	—	—	—
capacitance (pF)	220	26.6	75^k^	289.2	42.4	41.2	18.9	—	25.9	55.8
cell volume (pl)	22.6^m^	2.2^m^	2.9^l^	34.4	2.3^m^	2.3^m^	0.99^m^	1.4^m^	1.3^m^	2.9
SA/V ratio (pF/pl)	10	12	25.8^l^	8.44	18.3	18.2	19.1	—	19.9	19.2
t-tubular system	no	no	no^k^	yes	no	no	no	no	no	no^n^

Data are means but s.e.m. (when known) has been left out for clarity. An example from each taxa provided in figure 1 is given here. A dash means no data are available.

^a^*Lampetra fluviatilis* [26].

^b^*Danio reiro* [27].

^c^*Rattus norvegicus* [28].

^d^*Trachemys scripta scripta* [29].

^e^*Varanus exanthematicus* [30].

^f^*Python bivittatus*, D. Abramochkin 2021, unpublished observation.

^g^*Alligator mississippiensis,* B. Smith, D. Crossley and H. Shiels 2014, unpublished observations.

^h^*Meleagris gallopavo domesticus* [31].

^i^*Coturnix japonica* [32].

^j^*Rana esculenta* [33].

^k^*Rana catesbiana* [34].

^l^Derived from cell length and width assuming an elliptical cross-sectional area.

^m^Derived from cell capacitance (pF) following method of Vornanen [35].

^n^*Coturnix japonica* [16].

## 2. Myocyte morphology, architecture and excitation–contraction coupling

The strength and rate of heart contraction is controlled by excitation–contraction coupling and the cycling of Ca^2+^ at the level of the cardiomyocyte. Excitation–contraction coupling in all vertebrate myocytes proceeds from the action potential. Atrial and ventricular action potential waveform and the corresponding repolarizing currents (*I*_Kr_, *I*_Ks_, *I*_to_) have recently been characterized together for the first time in an adult bird (Japanese quail) [16]. Resting heart rates for these birds range between 318 and 530 beats min^−1^ [45,46] which is comparable to small rodents (mice/rats) and clearly depends on rapid/early ventricular repolarization [17]. However, the shape of the quail action potential demonstrates a plateau phase [16] which is more characteristic of mammals with slower resting heart rates (guinea pig/rabbit [47]) and fish [48]. The prominent action potential plateau is owing in part to the large influx of Ca^2+^ (*I*_Ca_) through voltage-gated L-type Ca^2+^ channels (LTCCs) in mammals (e.g. rabbit [47]), the quail [32] and other bird cardiomyocytes [36]. Indeed, a recent comparative study of cardiomyocyte ionic conductance across vertebrates shows bird ventricular myocytes have larger current densities than mammals when measured under similar conditions [49]. In another comparative study, ventricular action potential waveform across mammals of different sizes/heart rates showed *I*_Ca_ amplitude increased with increased body size. The authors suggested this reflects constraints imposed by the maintenance of excitation–contraction coupling in larger hearts [50]. Extending such studies to birds of different sizes, and birds with fast heart rates but large amplitude *I*_Ca_ would be very informative.

**Figure 3.**
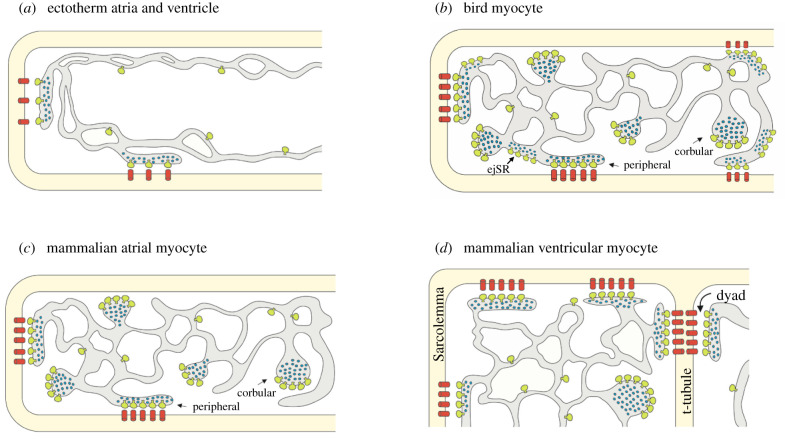
Schematic of the ultrastructural organization of sarcolemmal and SR membrane systems and their couplings in (*a*) an ectotherm (fish, amphibian, non-avian reptile) atrial or ventricular myocyte, (*b*) a bird ventricular myocyte, (*c*) a mammalian atrial myocyte (N.B. atrial myocytes from large mammals contain t-tubules [51]), and (*d*), an adult mammalian ventricular myocyte. Schematic shows sarcolemmal membrane containing L-type Ca^2+^ channels (LTCC, red) coupled at the periphery of the cell to the intracellular junctional SR (jSR) membrane system containing ryanodine receptors (RyRs), which cluster to form calcium release units (CRUs, pale green). CRUs are shown as a single RyRs for clarity but between 14 and 100 RyRs cluster together to form a CRU depending on the tissue and the species [38,52]. In (*b*) and (*c*) CRUs can also exist in non-junctional SR, as corbular SR (cSR) or extended-junctional SR (ejSR). These central CRUs facilitate the centripetal propagation of the peripheral Ca^2+^ signal. In (*d*) peripheral couplings (PCs) form at the surface sarcolemmal and dyadic couplings form along t-tubules facilitating synchronous Ca^2+^ release throughout the wider myocyte. Ca^2+^ inside the SR is illustrated by blue dots. For clarity, all other organelles are omitted from this schematic. Figure is adapted from [39] and amended with permission from Dr Gina Galli (original artist). (Online version in colour.)

The Ca^2+^ that enters via LTCCs induces further Ca^2+^ release from the intracellular stores of the sarcoplasmic reticulum (SR), in a process called Ca^2+^-induced Ca^2+^ release (CICR). The degree of CICR varies across vertebrates, being greater in birds and mammals than ectotherms [39]. Ca^2+^ is released from the SR into the cytosol through ryanodine receptors (RyRs), which cluster to form structures called CRUs within junctional regions of the SR membrane (jSR) (figure 3). Transsarcolemmal Ca^2+^ influx and SR Ca^2+^ release together form the rising phase of the cytosolic Ca^2+^ transient which activates the contraction of the myofilaments. Myocyte contraction ends when cytosolic Ca^2+^ levels fall owing to Ca^2+^ being removed from the cell via the sarcolemmal Na^+^–Ca^2+^ exchanger and being pumped back into the SR by sarco(endo)plasmic reticulum Ca^2+^ ATPase (SERCA) pumps located in the non-junctional or ‘free’(f) regions of the SR membrane.

The relative importance of transsarcolemmal-derived Ca^2+^ versus SR-derived Ca^2+^ in generating the Ca^2+^ transient varies across vertebrates with the relative proportion of the former generally dominating in ectotherms, and the latter generally dominating in adult endotherms (see [39] for review). The large surface-area-to-volume ratio of the bird cardiomyocyte (table 1, figure 2 and [16,32]) means transsarcolemmal Ca^2+^ influx can rapidly raise intracellular Ca^2+^ levels in the periphery of the thin cardiomyocyte, in-line with observations from ectothermic vertebrates [39,53]. However, unlike most ectothermic vertebrates, excitation–contraction coupling in birds also relies heavily on SR Ca^2+^ release [16,32] which amplifies the transsarcolemmal Ca^2+^ signal through CICR leading to stronger faster contractions [32].

Ca^2+^ diffusion is too slow to activate a coordinated and synchronized release of SR Ca^2+^ across the wider mammalian myocyte [54]. Adult mammalian ventricular myocytes get around this problem by having a t-tubule network comprised of invaginated surface sarcolemma which brings LTCCs into apposition with more centrally located SR membranes containing CRUs forming couplings called dyads [51,55–58]. The extent of the t-tubular network in mammalian atrial and ventricular myocytes depends on myocyte width (or cross-sectional area) reinforcing the role morphological architecture has on the organization of cellular Ca^2+^ cycling. The t-tubular network is mostly absent in narrow spindle-shaped sinoatrial nodal cells [59] and in narrow atrial cells of rodents and small mammals, but it is present in the wider atrial myocytes of larger mammals (e.g. horse, cow, human [41,51,60]). Thus, it cannot be excluded that t-tubules exist in very large bodied birds; however, they are not found in the myocytes from the turkey [31]. T-tubules are present in all adult mammalian ventricular myocytes where they govern temporal and spatial properties of the ventricular Ca^2+^ transient [41,51,57]. They are absent in developing and neonatal mammalian hearts. As the size of the myocyte grows postnatally to facilitate heart growth, t-tubules appear coincident with increased SR complexity, forming dyadic couplings and facilitating stronger contractility [61]. Bird cardiomyocytes do not hypertrophy during post-hatch development, rather the narrow, elongated architecture persists but is associated with changes in the intracellular architecture of CRUs within the SR membrane. An increase in the amount and structural organization of internal CRUs has been documented via electron microscopy at hatch and during post-hatch growth in sparrow [62] and chicken heart [63] and is concomitant with an increased reliance on SR cycling [64].

### (a) Peripheral and non-peripheral couplings and Ca^2+^ release from the sarcoplasmic reticulum

CICR can only occur when LTCCs in the sarcolemmal membrane and CRUs in the SR membrane are in close apposition forming couplings [61,65] (figure 3). All vertebrate myocytes have ‘peripheral couplings' (PCs) (e.g. rabbit [66], chicken [38,63], finch [65], anole lizard [67], frog [68] and fish [69]) comprised of CRUs in the peripheral jSR, directly opposed to sarcolemmal LTCCs [61,65] (figure 3). Ca^2+^ released from the SR at these PCs must diffuse centripetally to activate the myofilaments and to initiate the release of more centrally located CRUs. In ectotherm myocytes (i.e. fishes (rainbow trout [70]) and anole lizard [65]), this occurs slowly despite their thin morphology, as the peripheral Ca^2+^ signal falls rapidly the further it travels from the periphery [70]. Here, Ca^2+^ is taken up by peripherally located myofilaments [35,69,71] and adjacent mitochondria (see the contribution by Birkedal *et al.* [43] in this special issue), or can be buffered in the cytosol [72]. Bird myocytes [73] and mammalian atrial myocytes [60,74] limit this attrition of the Ca^2+^ signal by having a large number of centrally located (i.e. non-peripheral) CRUs, formed in a region of the SR membrane known as the corbular SR (cSR). These central cSR RyR clusters are not associated with the surface sarcolemma [75], rather they are associated with the z-line [38,55,73] and are activated by Ca^2+^ released at the periphery or by Ca^2+^ released by neighbouring cSR CRUs. These central CRUs contribute to the global Ca^2+^ transient [55,61] and underpin excitation–contraction coupling in the absence of a t-tubule network in bird [38,65] and non-tubulated mammalian atrial myocytes [60,74,76].

Myocytes from birds with fast heart rates like finch and hummingbird have another type of SR membrane coupling system called extended-junctional SR (ejSR) [73,77] (figure 3*b*) that is not observed in chicken or mammalian heart [78]. The ejSR extends centripetally either continuously or discontinuously from PCs along the z-lines and contain closely packed CRUs that are thought to serve as a fast conduit for intracellular Ca^2+^ release in a manner analogous to the CRUs in the cSR [55,73,77]. The development of more extensive CRU organization in birds with faster (finch, hummingbird [73], sparrow [62]) compared with slower (chicken [73], ratite [78]) heart rates clearly illustrates the importance of architecture for function. Indeed, this point was eloquently summarized by Sommer in 1995: ‘The geometry of the SR in striated muscle is crucial for excitation–contraction coupling. It determines the vectors and time course of effective calcium displacements’ [62, p. 24]. An ultrastructure study of the tinamou cardiomyocyte would be fascinating in this regard. Tinamous are a basal bird lineage with limited flapping-flight capability, low aerobic capacity and the smallest heart-mass-relative-to-body-mass of any bird [25,79].

### (b) Importance of subcellular organization of Ca^2+^ release units

The idea that coupling of the Ca^2+^ signal from PCs to centrally located CRUs are key to strong and fast contractions in birds was first suggested by Jewett and Sommer more than 40 years ago [73] (and see [62]) and is supported by a host of comparative ultrastructure studies in birds (e.g. [63,65,73,77,78,80]). The location and frequency of CRUs within the SR are key for understanding the rate and strength of the propagating Ca^2+^ signal which determines the synchrony of excitation–contraction coupling [55,61,81]. The distance between PCs is probably too great for lateral activation along the sarcolemmal membrane of neighbouring PCs in most animals including birds and large bodied mammals, meaning that the peripheral Ca^2+^ signal moves centripetally (not laterally) to activate CRUs [65,76]. Recent simulation showed that at distances of 250 nm or greater, there was no impact of changing the distance between PCs on the activation of the cellular Ca^2+^ signal [38]. A few measurements of distances less than 250 nm between PCs have been observed in finch but not in the chicken heart [38,55,65] and thus perhaps at shorter distances, PC spacing in the sarcolemmal membrane could influence rate and strength of excitation–contraction coupling. Indeed, in rat atrial and ventricular myocytes PCs less than 100 nm apart have been documented to allow lateral propagation of the Ca^2+^ signal [82].

The z-lines of the sarcomere form the backbone of myofilament contraction and studies across birds show ejSR/cSR and z-lines align [55,73,77,80]. In ectotherms, PCs are also concentrated at the z-lines, ensuring Ca^2+^ diffusion to the more loosely organized internal SR release sites situated along the z-lines [65,69]. The distance between cSR CRUs along a z-line were shown to have a major impact on the rate of rise of the Ca^2+^ transient in a two-dimensional avian model. As the distance between CRUs was increased from 100 to 600 nm, activation time was slowed by approximately fourfold [38].

The complement of ejSR and/or cSR and the number of CRUs (assessed as RyR density) within the ejSR and/or cSR in different bird species correlates with cardiac performance when assessed via electron microscopy. When measured under the same conditions, the chicken left ventricle had a lower complement of cSR (0.39 ± 0.11 µm of cSR per cardiac muscle fibre cross section (µm^−2^), *n* = 78) and a lower density of RyRs per cardiac muscle fibre volume (52 RyRs µm^−3^) compared with finch left ventricle (0.6 ± 0.14 µm ejSR per fibre cross section (µm^−2^), *n* = 231, and 144 RyRs per fibre volume (µm^3^), respectively) [65]. Similarly, a high complement of ejSR and/or cSR was found in hummingbird myocardium [73] compared with birds with slower hearts such as chicken and pigeon [77]. Thus, structural and computational modelling studies emphasize the importance of geometry and physical distances between CRUs within non-tubulated myocytes for determining the amplitude and time course of the intracellular Ca^2+^ transient, which regulates the strength and rate cardiac pumping.

## 3. Functional studies of Ca^2+^ flux in the bird cardiomyocyte

There are only four functional reports of excitation–contraction coupling in adult bird cardiomyocytes [16,31,32,36] to date, but all support the schema derived from ultrastructure studies. Evidence of a large transsarcolemmal Ca^2+^ influx carried by LTCCs comes from electrophysiological studies on isolated cardiomyocytes from the turkey [31], finch [36] and quail [32]. In finch heart, the density of *I*_CaL_ was more than twice that of mammals (rat) when recorded under the same conditions [36] and although comparing across studies can be difficult (i.e. owing to differences in the intracellular and extracellular solutions used in patch clamp studies, differences in acclimation or experimental temperature, etc.), *I*_CaL_ density measured in bird studies is consistently greater than that reported for ectotherms (e.g. fish [83]; turtle [29]; lizard [30], and see recent comparative review [49]). The high-density *I*_CaL_ also corresponds well with reports of a large complement of LTCCs in the bird sarcolemma assessed with radio-ligand dihydropyridine-binding [31]. Given the greater surface area–volume ratio (table 1) and thus the large contribution of sarcolemmal influx to cytosolic ionic composition, this large *I*_CaL_ is set to prime the bird myocyte for a Ca^2+^ transient with a large amplitude and fast raising phase, which underlie the strong and fast contractions observed by Kim *et al*. [31] in turkey ventricle.

The L-type Ca^2+^ current (*I*_CaL_) is the trigger for CICR at PCs and the greater the amplitude of *I*_CaL_, the greater the release of Ca^2+^ from the SR [84]. In this way, *I*_CaL_ amplitude drives the gain of CICR in cardiomyocytes [85]. Thus, the large density *I*_CaL_ in birds will drive a large release of Ca^2+^ from the adjacent CRUs provided they are adequately coupled. Tight coupling between LTCC and CRUs in PCs has been reported in all structural studies of the bird myocardium, but only recently was this confirmed functionally. Using freshly isolated quail ventricular myocytes Filatova *et al.* [32] showed that *I*_CaL_ caused Ca^2+^ release from the SR and further that Ca^2+^ release from the SR impacted the inactivation kinetics of *I*_CaL_ thus demonstrating for the first time: (i) functional crosstalk in bird PCs and (ii) the high gain of CICR in bird ventricular excitation–contraction coupling. These functional studies confirm earlier reports from [^3^H]ryanodine binding studies which showed the density and Ca^2+^ sensitivity of bird (pigeon and finch) RyRs are similar to those of mammals (rat) [77]. Interestingly, crosstalk was not observed in quail atrial myocytes where the amplitude of I_CaL_ was considerably smaller [32]. The study was conducted at room temperature and because I_CaL_ is temperature-dependent, the authors point out that CICR would also occur in atrial myocytes at the body temperature of the quail [32]. Additionally, there may be a basal level of stimulation (e.g. adrenergic tone) that exists *in vivo* but is absent *ex* *vivo* that enhances atrial I_CaL_ conductance in quail heart.

The presence of functional crosstalk and CICR in quail cardiomyocytes align with ultrastructural and radio-ligand binding studies to provide a clear mechanism for a strong and rapid Ca^2+^ signal occurring in the periphery of the myocyte. Presently we lack dynamic imaging studies which show the propagation of the peripheral signal through ejSR/cSR to the centre of the bird cardiomyocyte. However, such studies have been performed on the elongated/spindle-shaped myocytes from mammalian atrial cells [60,86]. Interestingly, in rat atrial myocytes (which are not tubulated) the peripheral Ca^2+^ signal is not effective at triggering CICR from centrally located non-junctional RyRs under normal conditions. Enhancing the amplitude of *I*_CaL_, RyR sensitivity to the cytosolic Ca^2+^ trigger, or increasing the Ca^2+^ content of the SR, have each been shown to be sufficient to trigger centripetal Ca^2+^ propagation and a global Ca^2+^ transient in mammalian atrial myocytes [74,87]. This enhancement can be accomplished with sympathetic activation of the heart, and in fish myocytes, adrenergic stimulation has been shown to enable CICR (CICR is minimal in the unstimulated state) [53]. The large amplitude *I*_CaL_ and the large SR Ca^2+^ content (discussed below), combined with the organization of CRUs in the bird myocardium are all indicative of a rapid and near-uniform global Ca^2+^ transient during routine excitation–contraction coupling. However, this must be substantiated experimentally.

### (a) Implications of high sarcoplasmic reticulum Ca^2+^ content in bird cardiomyocytes

Cytosolic Ca^2+^ is returned to the SR by the pumping activity of SERCA which facilitates the strong and rapid contractions of the bird heart. SR vesicles from adult turkey ventricular homogenates demonstrated robust SERCA activity [88] that correlated with a rapid decay in the multicellular Ca^2+^ transient in turkey heart preparations [31]. Refilling of the quail SR following depletion with caffeine follows a similar time course as mammals [89,90] and was four times faster than fish [91] when compared under similar experimental conditions.

Similar to ectotherms [39], the bird SR is able to hold a substantial amount of Ca^2+^ without spontaneously releasing it. SR Ca^2+^ content grows during development in the chicken heart with late-stage embryonic chicken myocytes having a steady-state content of approximately 400 µmol l^−1^ Ca^2+^ [64]. This compares with approximately 425 µmol l^−1^ Ca^2+^ steady-state content assessed via caffeine application in adult quail ventricular myocytes [32]. These values are on par with steady-state ventricular SR Ca^2+^ content measured in ectotherms (200–500 µmol l^−1^ Ca^2+^) [92,93] and greater than that in mammals (60–100 µmol l^−1^ Ca^2+^) [39,94,95]. The maximal Ca^2+^ content (greater than 1000 µmol l^−1^ Ca^2+^) of the fish SR [92,93] greatly exceeds that of mammals (50–200 µmol l^−1^ Ca^2+^) when both are assessed by the application of 10 mM caffeine [94,96]. Maximal SR content has not been specifically studied in the bird heart, but levels reached greater than 750 µmol l^−1^ Ca^2+^ during 100 steady-state loading pulses when *I*_CaL_ was not of sufficient magnitude to trigger CICR in the quail atrium [32]. Thus, it would appear that cardiac SR Ca^2+^ storage capacity has been dramatically reduced during the evolution of the mammalian myocyte.

There are many possible reasons why the bird (and ectotherm) SR hold a larger quantity of Ca^2+^ than mammalian SR (for review see [97]). On the cytosolic face, the opening of the RyRs is triggered by cytosolic Ca^2+^ and despite similar [^3^H]Ryanodine binding affinities in bird and mammal [77], other (yet unknown) ligands may be important for sensitizing RyR opening in response to cytosolic Ca^2+^ in birds. Indeed, the activity of the RyR is regulated by many intracellular factors such as Mg^2+^, nucleotides, proteins and reactive oxygen species [98], none of which have been studied in the bird heart to date. In ectotherms, but probably not adult birds, the Ca^2+^ sensitivity of the RyRs [99], density of RyRs [100,101], number of RyRs in a CRU and the distances between CRUs also probably factor into CICR failure despite a large SR Ca^2+^ content [97]. However, as discussed above, a large enough *I*_CaL_ trigger should be sufficient to release SR Ca^2+^ in all elongated myocytes, except maybe in amphibians [53,68,102].

Perhaps the most interesting aspect of the large Ca^2+^ content in bird heart is that it does not cause spontaneous opening of the RyRs from the luminal side. In mammals, RyR opening is triggered by luminal Ca^2+^ which can potentiate the effects of cytosolic RyR activators [103–105]. In rat myocytes, Ca^2+^ waves indicative of RyR opening occurs when SR Ca^2+^ content exceeds a threshold of approximately 60–100 µmol l^−1^ Ca^2+^ [94]. The reason(s) why SR Ca^2+^ content can reach nearly ten times this amount in bird (and ectotherm) heart without spontaneous release is not known. Luminal Ca^2+^ sensing is strongly dependent on the SR Ca^2+^-buffering capacity (e.g. calsequestrin) and on interactions with luminal proteins (e.g. triadin and junctin) [98]. However, nothing is known about these proteins and how they might interact or how they might regulate RyRs in non-mammalian hearts, but clearly having a high SR Ca^2+^ storage capacity must be coupled to a low release sensitivity in birds and ectotherms. Release of the entire SR Ca^2+^ content (which approaches mM levels at maximal loads [92,93]) would be catastrophic to excitation–contraction coupling and induce toxicity (i.e. mitochondrial Ca^2+^ overload) and dysfunction (i.e. arrhythmias) often associated with cytosolic Ca^2+^ overload in ischemia–reperfusion scenarios [106,107]. As Ca^2+^ overload and errant SR Ca^2+^ release underlie many human cardiomyopathies, understanding how bird cardiomyocytes regulate SR Ca^2+^ storage and release could provide novel avenues for therapeutics.

## 4. Cardiomyocyte morphology, endothermy and regeneration

The narrow, elongated cardiomyocyte morphology dominates the animal kingdom including cephalopods [108] (table 1 and figure 2). In fishes and amphibians, this gross morphology has been associated with the Frank–Starling law of the heart and stretch regulation of cardiac output [109–111]. The sarcomeres of fish [112] and amphibian [113,114] myocytes are able to stretch further, and develop force at longer lengths than those of mammalian myocytes. However, despite very large stroke volumes, and thus large end-diastolic volumes [11], evidence from whole hearts and single cells from turkeys suggest bird active and passive length–tension properties are more similar to mammals (stiff) than to fish (compliant) [115]. Indeed, birds are thought to predominately modulate cardiac output via increases in heart rate with exercise [116], but this may be owing to near maximal stroke volumes at rest. Moreover, changing sarcomeric spacing akin to myocardial stretch had little effect on computed Ca^2+^ activation time in an avian heart cell model [38]. Thus, elgonated myocyte morphology does not appear related to enhanced length-dependent activation in the bird heart.

The elongated myocyte morphology is also associated with cardiac regeneration. Neonatal mammalian, embryonic bird, fish, urodele amphibian and reptile hearts [1,117,118] are all able to regenerate and all have an elongated myocyte morphology. Thus, it would be tempting to suggest a causative link. However, although hearts of 5-day chick embryos were able to regenerate, this ability was lost in 18-day embryos and in newly hatched chicks, indicating that despite maintained myocyte morphology from late stage embryo to post-hatch development, regeneration capability in birds is lost [118]. This loss of proliferation-potential in post-hatch bird myocytes is interesting, as it is another trait associated with endothermy [44,119]. Indeed, all birds are ectothermic *in ovo* and attain endothermy post-hatch [11,120]. Different species of bird attain endothermy at different time points during post-hatch development [7,11]. Precocial species that hatch feathered, active and able to find their own food, attain endothermic thermoregulatory capacity at hatch through the rapid development of the aerobic capacity to support increased energy demands [7,121]. In the precocial duck, heart mass almost doubles in the last 24 h before hatch, and in duck and chicken embryos, oxidative phosphorylation capacity of cardiac mitochondria also significantly increases in the last 24 h before hatch, presumably in preparation for endothermic energy demands [120,121]. In altricial species, endothermy develops post-hatch during nesting [120]. Such changes in cardiovascular capacity are not observed paranatally in closely related ectothermic species such as the American alligator [122].

The mechanisms linking endothermy and non-proliferating cardiomyocytes is an active area of research that has recently been linked to another key feature of cardiomyocyte architecture—polyploidy or genome duplication. By and large, all bird and mammal embryonic and neonatal cardiomyocytes, and most ectotherm cardiomyocytes (independent of age) are mononuclear and diploid [44,123,124]. During development, the endotherm heart grows by the expansion of cardiomyocyte number (hyperplasia). However, postnatally/post-hatch a large proportion of endothermic (but not ectothermic) cardiomyocytes that enter the cell cycle do not complete it resulting in endoreplication [44,124]. This produces myocytes with more than one copy of their diploid genome in a single nucleus or in multiple nuclei within a single cell. For example, polyploidization of cardiomyocytes in quail was shown to occur during the first 40 days post-hatch, and end by the time body growth is completed at 60 days [123]. This change in genome size and structure coincides with other aspects of myocyte maturation including increased SR complexity (CRUs in birds, t-tubules in mammals). Metabolic and hormonal remodelling associated with postnatal growth (mammals) or post-hatch endothermy and growth (birds) [119] result in increased mitochondrial metabolism and reactive oxygen species generation at the same developmental timepoint [7,120,121]. Increased ploidy may provide additional transcriptional output for protein biosynthesis in endothermic cardiomyocytes with high metabolic activity [44] in a trade-off with decreased capacity for proliferation [44,124,125]. This accords with higher incidence of polyploid in precocial species than in altricial birds of the same weight post-hatch, owing to increased functional cardiac load during development [123]. Additionally, in a comparative study of 31 species of adult birds, cardiomyocyte polyploidy is higher in species, and in cardiac chambers across species, that have increased cardiac work-loads [125]. Polyploidy does increase cell size by a small but significant amount in both birds and mammals [123,126,127]. However, this slight change in geometry is concordant with the structural changes underlying excitation–contraction coupling in both hypertrophied mammalian ventricular myocytes and in narrow bird myocytes. Thus, ploidy is not a function of myocyte size, rather, a consequence of myocyte metabolism. Further work is required to properly elucidate relationships between myocyte morphology, ploidy, regeneration and endothermy. However, birds have largely been ignored in this pursuit, and the ectotherm–endotherm transition in bird development may hold the key to understanding the balance between resource allocation in polyploid cardiomyocytes and reduced capacity to proliferate, compared with mononucleated diploid cardiomyocytes and the preserved capacity to proliferate.

## 5. Summary and perspective

The elongated cardiomyocyte of ectothermic vertebrates is internally remodelled with a superhighway of CRUs in post-hatch birds to increase cardiac force and cardiac frequency beyond that of their ectothermic ancestors. Clearly this strategy is equally as successful as the hypertrophied and t-tubulated myocytes of adult mammals for powering the robust cardiac function necessitated by endothermy. Indeed, the force generated per cross-sectional area of the ventricular wall is similar in mammals and birds [20], emphasizing the point that despite differences in cellular architecture the functional output of the bird and mammal myocardium is very similar.

However, detailed functional studies of bird cellular Ca^2+^ flux during excitation–contraction coupling are scarce, and knowledge of the spatial and temporal properties of the intracellular Ca^2+^ transient is lacking. These are necessary to improve our structural and functional understanding of the hearts of this group of endothermic vertebrates. Birds also comprise the only taxon that transitions from ectotherm to endotherm during development. Studies of their elongated myocytes throughout this transition are uniquely placed to shed insight into a key area of human cardiac research—the drivers of polyploidization at the expense of proliferation in heart regeneration.

Currently, one-in-seven (14%) of the world's bird species are threatened with extinction (greater than 4000 species) [128] and so cardiac diversity in this important group is being lost to science at an astounding rate. Thus, structural, physiological, and molecular/genomic studies of bird hearts must be coupled with urgent conservation to ensure this precious resource to science and global biodiversity is not lost.

## Data Availability

This article has no additional data.

## Glossary

altricial: birds that hatched unfeathered, have poor metabolic heat balance, and require significant parental investment during maturation in nest
*Ca^2+^ release units* (*CRUs*): cluster of ryanodine receptors in the sarcoplasmic reticulum (SR) membrane that open to allow Ca^2+^ release
*corbular SR* (*cSR*): specialized ‘flask-like’ structures within the SR membrane that contain CRUs not associated with sarcolemmal membrane; cSR is found in mammalian atrial myocytes and avian myocytes
ectothermy: where resting body temperature follows ambient temperature because the organism is unable to retain metabolic heat
endothermy: ability to raise body temperature above ambient while at rest through retention of metabolically derived heat
*extended-junctional SR* (*ejSR*): specialized form of jSR found in birds with rapid heart rates; ejSR extends from jSR at the cell periphery into the centre of the myocyte containing CRUs not associated with the sarcolemmal membrane
free SR: non-junctional SR that contains SERCA pumps and forms the bulk of the SR membrane
*junctional SR* (*jSR*): portion of the SR membrane containing CRUs that forms couplings with the sarcolemmal membrane
non-avian reptiles: the monophyletic group that includes reptiles, also includes birds (figure 1); thus, non-avian reptile is used in modern cladistics, in reference to the group of animals traditionally described as reptiles (such as turtles, lizards, snakes, crocodiles) but not including birds
peripheral couplings (PCs): coupling of jSR and sarcolemmal membranes at the cell surface where Ca^2+^-induce Ca^2+^ release occurs
ploidy: reference to the number of complete sets of chromosomes contained within a cell; typical cells are diploid with two copies; polyploidy can occur owing to multiple copies of the chromosome set within a single nucleus or by having multiple nuclei within a single cell
precocial: birds that are hatched with feathers, an established heat balance, and can procure food by themselves
